# Breast Cancer Patient Attitudes Towards Oncology Drug Costs in Ireland

**DOI:** 10.3390/curroncol33030161

**Published:** 2026-03-12

**Authors:** Matthew Cronin, Ruth Kieran, Clara Steele, Katie Cooke, Seamus O’Reilly

**Affiliations:** 1 Cancer Research@UCC, University College Cork, T12 CY82 Cork, Ireland; 118475496@umail.ucc.ie; 2 Department of Medical Oncology, Cork University Hospital, T12 DC4A Cork, Ireland; kieranr@tcd.ie (R.K.); 118731715@umail.ucc.ie (C.S.); katie.cooke@hse.ie (K.C.)

**Keywords:** cost, drug, breast cancer, immunotherapy, targeted therapy, health economics, cost reduction

## Abstract

**Background:** Oncology medication costs are increasing internationally; patient attitudes towards these costs remain unclear. **Methods:** A three-part cross-sectional questionnaire was distributed to patients with breast cancer to determine their attitudes towards oncology medication costs and to explore potential patient-acceptable methods to reduce these costs. **Results:** A total of 321 patients were eligible for inclusion and 180 fully completed the questionnaire (56.1% response rate). Overall, 67.8% (N = 122/180) of patients found the costs presented in the questionnaire to be unacceptable. 92.2% (N = 166/180), 87.8% (N = 158/180) and 68.9% (N = 124/180) of participants found the costs of pembrolizumab, palbociclib and trastuzumab respectively to be unacceptable. A total of 72.8% (N = 131/180) of patients indicated that they would like to be better informed about the societal costs of their cancer treatment and 81.1% (N = 146/180) of patients believed that reducing the costs of cancer treatment to society is important. There was a statistically significant difference in patient desires to be better informed of societal drug costs between those with early-stage breast cancer and those with metastatic disease (75.8% vs. 47.4%, χ^2^ = 6.923, *p* = 0.009). **Conclusions:** These findings indicate that many Irish patients with breast cancer find the societal costs of oncology medications to be unacceptable, and many patients have a desire to be better informed of these costs.

## Simple Summary

The cost of cancer medications is rapidly increasing, which presents a significant problem for patients and healthcare systems. Limited prior research has investigated patient attitudes towards the societal costs of cancer treatment and towards potential methods used to reduce these costs. This study found that a majority of patients with breast cancer found the societal costs of many cancer medications to be unacceptable. Furthermore, most patients indicated that they would like to be better informed of the societal costs of their treatment. These results further highlight the need to reduce drug costs and a potential desire amongst patients to be better informed of treatment costs. This study also identified several cost-reduction methods which were acceptable to many patients. This may help inform future policy decisions at international levels to ensure legislation reflects patient preference. Future studies should seek to investigate patient attitudes towards drug costs for other malignancies.

## 1. Introduction

The field of oncology has undergone rapid advancement in recent years. Both academic and industry-led research have provided people with a diagnosis of cancer more access to effective treatment options. Whilst this increase in available treatment options has been accompanied by an improvement in patient outcomes [1,2], the costs associated with these new treatments have also increased, limiting their availability and potential impact, which has resulted in financial hardship for patients even in affluent countries [3]. Annual global healthcare expenditure on pharmaceuticals is projected to exceed €2.2 trillion by 2028 [4]. Of this expenditure, oncology medications are forecast to approach €400 billion per year over the same period [5]. The situation in Ireland reflects this global trend. A recent report found that annual pharmaceutical expenditure in Ireland reached an all-time high of €2.54 billion in 2021 [6]. Furthermore, a report conducted by the Health Service Executive (HSE) on pharmaceutical spending indicates that “high-tech drugs” and “high-cost drugs” (of which oncology medications are key members) were two of the leading causes of this increasing expenditure [7].

Internationally, patients often bear much of the out-of-pocket costs of their cancer treatment [8] and are therefore acutely aware of these costs. This differs to models employed in many European countries, e.g., Germany, the United Kingdom and Ireland, where medications are largely reimbursed by governmental bodies [9]. In contrast, in Ireland, most oncology medications are subsidised under the Oncology Drugs Management System (ODMS), Primary Care Reimbursement Scheme (PCRS), local hospital budgeting or the Drug Payment Scheme. Under these programmes, the maximum out-of-pocket cost for patients is capped at €80 per family per month [10], even for drugs that may cost thousands of euros. This lessens the financial burden on individual patients and distributes the cost across society. Given that Irish patients are rarely exposed to the total costs of their treatment; their attitude towards growing prices remains unclear. Few existing studies have assessed patient attitudes towards the societal costs of cancer treatment [11,12,13,14,15]. Those studies which have generally present hypothetical clinical scenarios to patients. They then ask patients to indicate what cost they believe would be appropriate for a given treatment in each scenario. Whilst this provides some information on patient attitudes towards treatment costs, it makes interpreting them in the context of real-world prices somewhat difficult. It also makes discerning patient attitudes towards out-of-pocket costs as opposed to societal costs more challenging. For example, several studies have found that patients with a diverse range of malignancies are willing to pay more than $90,000 for cancer treatment when not specifically placed in a societal context [11,12,13]. However, one German study which explored preferences of patients with melanoma on healthcare spending in a societal context found that they more frequently allocated €100,000 of healthcare funding towards public health measures as opposed to spending on cancer medication [14]. Given the heterogeneity and lack of existing literature in this area, it remains unclear what patients’ attitudes are towards the societal costs of cancer medications.

In 2009, the American Society of Clinical Oncology released a seminal guidance statement outlining the economic issues facing cancer care provision [16]. In this statement, patient–physician cost discussions were endorsed as an important element of high-quality care. The report also highlights a paucity of research on the influence of societal costs on patient–physician communication. Despite this report being published over a decade ago, few studies have explored the relevance of societal costs to patient–physician communication. Instead, much existing research has focused on the direct out-of-pocket costs to patients [17,18,19]. One of the few studies conducted in this area found a majority of patients believed the societal costs of cancer care were concerning [20]. Despite these concerns, just 38% of participants indicated that they would like to discuss societal costs with their doctor. Another study found that over half of patients with a cancer diagnosis did not want their doctor to consider societal costs when making medical decisions [21]. These studies may indicate that cancer patients generally do not wish to engage in societal cost discussions. However, it is important to note that these studies were conducted in the United States, a country which employs predominantly privately provided healthcare delivery.

Increasing oncology drug costs present a significant challenge to clinical practice and to the sustainability of healthcare which is particularly pronounced in low- and middle-income countries [22]. Many methods have been proposed to reduce the growing costs of cancer care. The World Health Organisation, for example, has advocated for increased cost transparency to improve the affordability of cancer medications [23]. Other proposed methods include the use of less expensive alternatives such as generics or “biosimilars”. Few studies have explored patient attitudes towards a broad variety of these cost-reduction methods. One such study which explored patient attitudes towards cost-reduction methods found that 88% of participants were concerned about the costs of cancer care; however, there was little agreement in how these costs could best be reduced. Only one cost reduction method, namely generic substitution, had a majority of participants (58%) believe it would be a preferred method for cost-reduction [20]; however, this study was conducted in the United States. Patient preferences of cancer cost-reduction methods remain unclear in European and predominantly publicly funded healthcare systems. Determining patient attitudes towards cost-reduction methods may help to inform future policy discussions at national and international levels.

## 2. Materials and Methods

A cross-sectional survey was distributed at two university hospital outpatient breast cancer clinics in the South of Ireland, namely Cork University Hospital (CUH) and The South Infirmary Victoria University Hospital (SIVUH). Questionnaires were distributed from 1 July 2024 to 20 December 2024. A three-part questionnaire was designed by the study authors to assess patient attitudes towards societal oncology medication costs and cost-reduction methods. Several sections of the questionnaire built upon previous work; however, significant components were designed by the study authors. All sections underwent multiple revisions by a consultant medical oncologist, a specialist registrar in medical oncology and a specialist oncology pharmacist. A copy of the questionnaire can be found in the Supplementary Materials, Text S1. This study focuses on patients with breast cancer to provide a homogeneous patient cohort. As the aims of this study are primarily observational and hypothesis-generating, the sample size estimation was based on what was feasible at the two recruiting sites within a reasonable time frame as well as the sample size achieved in other limited research studies done in this area [20].

Patients were eligible for inclusion in the study if they had a diagnosis of breast cancer, were attending the breast cancer outpatient department and were aged > 18 years old. Patients were excluded if they were pregnant, aged < 18 years old, had a cognitive impairment, were unable to read or write, unable to complete the survey due to high burden of cancer symptoms/acute distress, had an Eastern Cooperative Oncology Group performance score of 3 or more, or those otherwise inappropriate to approach (in opinion of outpatient staff on day of appointment). Both male and female patients were eligible for inclusion. Convenience sampling was used for questionnaire distribution. Convenience sampling was used in this study for two main reasons. Firstly, the study authors aimed to ensure inclusion of a wide range of study participants who were indicative of the patient cohort attending outpatient breast cancer clinics in the South of Ireland. Secondly, due to a lack of a centralised patient data repository at the two recruitment sites, we were unable to use a more robust sampling technique such as probability sampling. Convenience sampling represented a feasible and effective sampling method for our study aims. Consent was implied by completion and return of the questionnaire.

Patients meeting the inclusion criteria during the study period were identified during outpatient medical oncology clinics. The lead author approached and offered the questionnaire to eligible patients. Participants completed the questionnaire in the outpatient waiting area. When completed, participants placed the questionnaire into a secure box in the outpatient waiting area. All fully completed questionnaires were uploaded to a password-protected computer for data analysis. The numbers of patients who were offered the questionnaire and who fully completed it were recorded. Any questionnaires that were not fully completed were not included in the final analysis. Ethical approval was received from the Clinical Research Ethics Committee of the Cork Teaching Hospitals on 12 February 2024 (ECM 6 (a) 5 March 2024).

The first section of the questionnaire consisted of non-identifiable demographic questions adapted from previous studies [20,24]. The second section contained elements from an existing survey [20] and inquired about general patient attitudes towards personal and societal oncology drug costs, using Likert scales. Patients were also asked to provide their views on potential methods to reduce the societal costs of oncology medications.

In the final section, the costs of eleven commonly prescribed breast cancer treatments, outlined in List 1 below, were presented to participants. Participants were first asked if they had been treated with any of the listed medications. They then indicated if the costs presented were more or less expensive than expected and finally, they indicated the acceptability of each cost. Participants were also asked about their overall views of the costs presented in the questionnaire. This third section of the questionnaire was designed entirely by the study authors. The costs used in the survey were chosen to be reflective of the costs of those medications in the year of questionnaire distribution (2024). The reason for this choice was that the aim of our survey was to explore patient attitudes towards contemporary cancer drug costs. An explanation of the cost calculations for the costs used in the questionnaire can be found in the Supplementary Materials, Tables S1–S4.

### List 1—Drug List

- Oral therapy: tamoxifen, anastrozole and palbociclib.
- Intravenous-targeted and immunotherapy: trastuzumab, pertuzumab, pembrolizumab and atezolizumab.
- Chemotherapy: paclitaxel, doxorubicin, docetaxel and cyclophosphamide.

Data were compiled and analysed using IBM SPSS Statistics 29.0. Descriptive statistics were used to analyse demographic data, general and specific attitudes towards drug costs and drug-cost-reduction methods. Due to the observational and exploratory nature of this study, we chose to use Mann–Whitney U and Chi-square tests to investigate associations between participant characteristics and participant attitudes towards oncology drug costs as well as associations of patient desires to be better informed of the societal costs of their cancer treatment. These methods do not allow for adjustment for covariates and as such may contribute to confounding.

Participant characteristics investigated in the Mann–Whitney U and Chi-square tests included participant age, education level, annual household income, time from diagnosis, disease and use of expensive medications (defined as a cost > €10,000). Educational level was recoded from a five-item variable to a binary variable. It was dichotimised into those without a degree level education and those whose highest level of education was a degree, diploma or certificate. Those who reported that their highest level of education was “Any Primary” or “Any Secondary” were coded into the “No Degree” category. Those who reported their highest educational attainment as either a “Diploma/certificate”, “Primary University Degree” or “Postgraduate Degree” were coded into the “Degree/diploma/certificate” category. Due to the limited research in this area, the participant characteristics chosen by the study authors for investigation were selected as they were hypothesised theoretically to be relevant to patient cost acceptability and patient desires to discuss treatment costs. Characteristic selection was also informed by available existing research [16].

## 3. Results

Three hundred and twenty-one patients were offered the questionnaire, of these 180 fully completed the questionnaire, yielding a response rate of 56.1%. Participant demographics are presented in Table 1. Of the eleven medications presented in the questionnaire, the three most commonly prescribed were anastrozole at 48.9% (N = 88/180), tamoxifen at 47.2% (N = 85/180) and paclitaxel at 15% (N = 27/180).

### 3.1. Attitudes Towards Societal Medication Costs

Fifty nine percent (N = 106/180) of participants indicated that they understood the societal costs of their treatment. Seventy three percent (N = 131/180) of patients indicated that they would like to be better informed about the societal costs of their cancer treatment. Eighty one percent (N = 146/180) believed that reducing the costs of cancer care to society is important.

**Figure 1:**
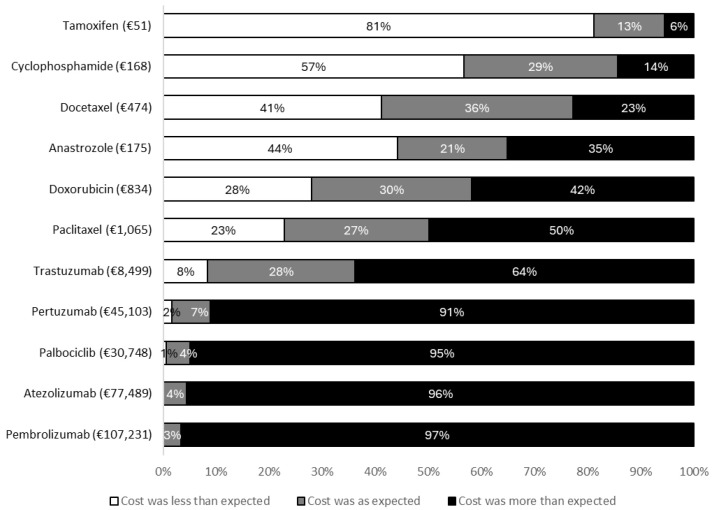
Patient expectations of drug costs.

After viewing the costs in the questionnaire, 87.2% (N = 157/180) of patients reported being either surprised or very surprised by the prices presented. Eighty one percent (N = 146/180) believed the costs presented were higher than they had expected. The degree to which the prices presented in the questionnaire agreed with patient expectations are shown in Figure 1.

**Figure 2:**
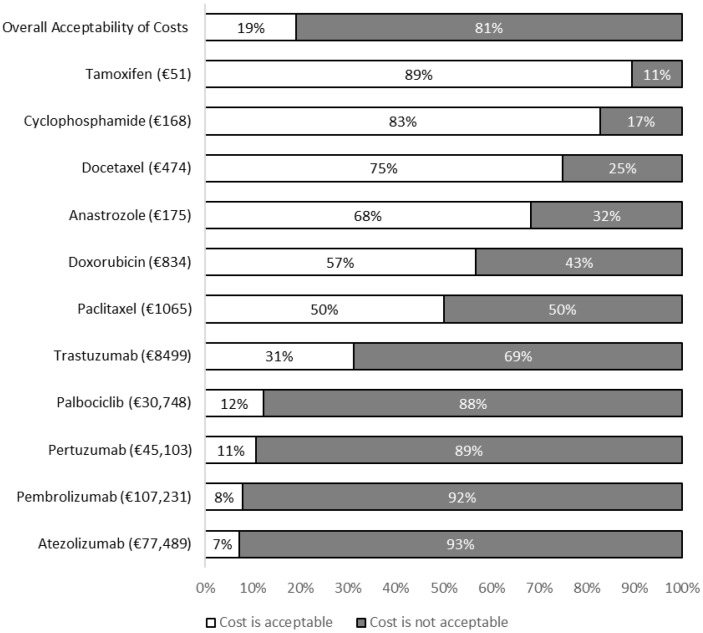
Proportion of participants finding the costs presented to be acceptable or unacceptable.

Nineteen percent (N = 35/180) of respondents believed that overall, the costs presented in the questionnaire were acceptable. The proportions of patients finding the costs presented in the questionnaire to be acceptable are presented in Figure 2. Chi-square and Mann–Whitney U tests were used to determine characteristics associated with patients identifying the costs presented in the questionnaire to be acceptable or unacceptable (Table 2), and the desire to be informed of them Table 3.

### 3.2. Attitudes Towards Societal Cost-Reduction Methods

**Figure 3:**
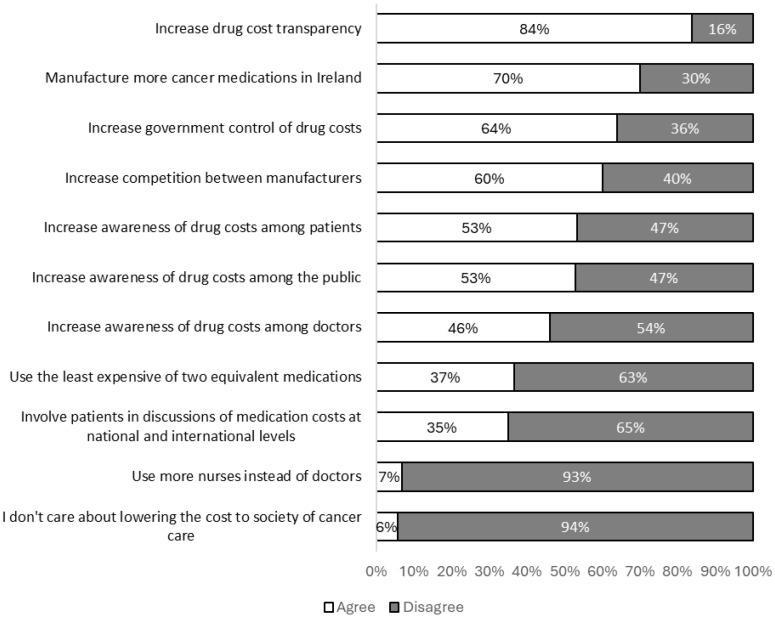
Patient preferences for control of costs of cancer care.

The most popular methods among participants to reduce the societal costs of cancer care were increased drug cost transparency, increased production of cancer medications in Ireland and increased governmental control of medication costs. Specific attitudes towards cost-reduction methods are shown in Figure 3.

## 4. Discussion

This study investigates the attitudes of patients with breast cancer towards the societal costs of cancer care and societal cost-reduction methods. Patients found the societal costs presented in the questionnaire to be surprisingly higher than expected. Many patients indicated a desire to be better informed of these costs. Statistical differences in patient desires to be informed of costs were observed between those with early stage and metastatic disease as well as those with a degree/certificate/diploma level education versus those without. Participants found many of the proposed cost-reduction methods to be acceptable, which adds to existing international work on potential cost-reduction methods [20,25].

Fewer than one in five participants found the costs of the four most expensive medications in the questionnaire to be acceptable. In contrast, most patients found the costs of the four least expensive medications to be acceptable. The least expensive medications in the questionnaire were also generally the most commonly prescribed in the study population. These findings may indicate that it is the costs of an expensive, infrequently prescribed group of medications which patients find to be unacceptable. It is important to note that the costs presented to patients in our questionnaire were derived from UK and Irish cost data and so may be more applicable to Irish/European contexts.

This is one of the first studies of its kind which explicitly presents patients with real-world oncology drug costs to determine their attitudes. In one previous study conducted in the Netherlands, approximately 30% of patients with cancer believed that an annual cost of more than €240,000 was acceptable [11]. In a large, multi-country study examining the costs that patients, the general population and caregivers deemed should be spent on treatment in exchange for an extra year of life, 40% of those in European Union countries indicated that up to €200,000 or more should be spent and 24% of those in the United States believed $200,000 plus should be spent [12]. One study conducted in the United States found that a quarter of breast cancer patients were willing to pay at least $90,000 for a “hopeful gamble treatment” [13]. In the context of this study a “hopeful gamble treatment” refers to a treatment which has a chance of increasing survival by a substantial amount. These findings are contrary to those of our study. Higher proportions of patients in our study found the costs of the most expensive medications to be unacceptable even when the costs were significantly lower than those described in international studies.

Two thirds of German patients with melanoma reported they were willing to spend up to €100,000 for the immunotherapy treatment ipilimumab [14]. However, in the same study, when presented to patients in a societal context, participants chose to allocate €100,000 from health funds at a higher rate towards skin screening (46%) and primary prevention (46%) as compared to ipilimumab (4%). An additional German study also observed similar findings [15]. When placing patients with melanoma in the hypothetical position of a treating physician, half of the patients opted to spend money on combination immunotherapy which could otherwise be used for prevention measures against cancer despite the high costs associated with combination immunotherapy. In contrast, when presented in a societal perspective, 73% of patients with melanoma favoured spending €150,000 from the health fund on skin cancer screening for early cancer detection rather than on immunotherapy or palliative care. These findings indicate that patients with cancer may have different views towards healthcare costs at a societal level compared to an individual care level. The cost of the most expensive medication included in our study was approximately €110,000 per year and just 8% of our study participants found this cost to be acceptable. Taken together, these findings may explain the relatively lower rate of cost acceptability amongst participants in our study compared to the rates observed in other international studies.

Almost three quarters of patients indicated that they would like to be better informed of the societal costs of their cancer care. Our study also found a statistically significant difference in patient desires to be better informed of societal drug costs with respect to disease stage and education level. There was a higher proportion of patients with early-stage breast cancer who wished to be better informed of costs than those with metastatic disease. Given the hypothesis-generating nature of this article, it remains unclear how cost information could best be distributed to those patients who most wish to be informed of costs. Our results differ from existing research which identified no association between patient desires to discuss societal costs with their doctor and stage of disease [20]. Previous studies exploring patient attitudes of societal costs found that most patients do not want their doctor to discuss the societal costs of care with them or for their doctor to consider societal costs when making medical decisions [20,21,26]. Interpreting our findings in this context may indicate that whilst patients do desire to be informed of societal costs, they do not wish for their doctor to provide this information. It is important to realise that these previous studies were conducted in the United States, so any inferences or extrapolations should be made with caution. Furthermore, exploring the potential means by which such information could be communicated was not an aim of our study. Further cross-sectional research is needed to explore patient perspectives on how best to supply suitable patients with this information.

Six cost-reduction methods were acceptable to more than 50% of patients in our study. In a previous study investigating patient attitudes towards cost-reduction techniques, this level of agreement was achieved for just one method: the use of generic substitution [20]. Additionally, in this previous study, just 33% of participants identified government control of cancer drug prices as a potential method to reduce societal costs. Patients in our study selected increased governmental control of drug costs twice as frequently, making it the third most popular cost-reduction method overall. Increasing government control of drug costs and increased drug cost transparency were both popular cost-reduction methods among patients. These two methods are rather general approaches to cost reduction and so may be logically applied to other healthcare systems that receive public funding.

Over half of participants indicated that increasing cost awareness among patients could help reduce societal costs; however, just 35% of participants selected “involving patients in medication cost discussions at national or international levels” as a potential cost-reduction method. This may indicate that whilst patients believe engagement with societal costs at an individual level is appropriate, they see little role for involvement at a collective policy-making level.

### 4.1. Strengths and Limitations

A key strength of this study is its broad inclusion criteria. This enabled participants from broad socioeconomic backgrounds and patients at varying points in their disease trajectory to take part. Patients within one year of their diagnosis through to those almost 30 years from original diagnosis were included. Furthermore, participants with a variety of education levels participated in this study.

A key limitation of this study is the possibility of confounding, as multivariable analysis was not performed. Given that our aims were observational and given the hypothesis-generating and exploratory nature of this study, the authors decided to use Chi-square and Mann–Whitney U tests without multiple testing adjustment.

Another limitation is the possibility of non-response bias, as we achieved a response rate of 56.1%. Reasons for non-response could include a high burden of disease or sociodemographic differences in participants. As described, the views of patients with metastatic disease were observed to differ to those of patients with early-stage disease. Therefore, non-response due to disease burden may mean that this important subgroup is underrepresented which may affect our results. Furthermore, this study was conducted in the same setting as the participants’ usual clinical care, and participants were recruited by their treating clinical team, therefore social desirability bias is also difficult to exclude. The stability of social desirability bias has been previously shown to be influenced by educational history [27] and this may partially account for the association between desiring to be better informed of drug costs and education. This association between desirability of cost discussions and education may also be related to the fact that this study was conducted in Ireland, a high-income country. As a result, it is possible that there is a greater proportion of educated individuals who have a greater awareness of current issues surrounding the costs of cancer medications. As such, they may not feel it necessary to be better informed of such costs. The only comparable study available in this area did not report such an association [20].

It is also important to note the considerable complexity of explaining the relative efficacy of different cancer medications in the context of a questionnaire. As such, in our questionnaire, we simply presented participants with a number of generally well-established cancer medications. We described their typical method of administration and provided an estimate of the cost of each medication for a given duration of treatment. There is therefore a risk that participants’ acceptance of different drug costs may be based primarily on the cost alone, which may represent a source of bias.

An important limitation of this study is its generalisability. Whilst male breast cancer patients were eligible for inclusion in this study and were approached during study recruitment, none fully completed the questionnaire. This study also included low proportions of patients with metastatic disease. Similar studies have achieved inclusion rates of patients with metastatic disease as high as 33% [19], compared to 10% achieved in our study. Our findings indicate that patients with metastatic disease have differing views on societal drug costs compared to those with early-stage disease. Given the low participation rate of patients with metastatic disease, this may affect our results. Furthermore, medication costs vary greatly between countries. Healthcare systems around the world differ in their levels of public and private sector involvement. Therefore, applying the results of this study to other countries and healthcare systems may be difficult and should be done with care. This limitation is further evidenced by the differing results in comparison to research conducted in the United States. Finally, this study did not collect data on the insurance status of participants. We recognise that this may be a significant limitation of the study, as it may confound the associations observed in our study. However, given the hypothesis-generating nature of this study, insurance data was not measured. Had insurance data been included, it may also have enhanced comparisons to other healthcare systems.

The study questionnaire did not undergo validity and reliability testing. Furthermore, it did not undergo focus group testing prior to distribution. The questionnaire did however undergo several revisions by a consultant medical oncologist, a specialist registrar in medical oncology and a specialist oncology pharmacist. The authors recognise this as a limitation of the study.

Additionally, this study utilised convenience sampling. The authors recognise that this limits the study’s generalizability; however, convenience sampling represented an effective sampling method within the constraints and aims of this study. Future research should aim to use more robust sampling techniques such as probability sampling.

### 4.2. Health Policy Implications and Future Research

The results of this study have several potential health policy implications. Firstly, our study highlights that several cancer medications are unacceptable to a significant proportion of patients with breast cancer. This adds weight to the global discussion surrounding cancer medication costs as it provides a patient perspective. Secondly, many patients with breast cancer in our study wished to be better informed of the societal costs of their cancer care. This may highlight a need for healthcare systems similarly structured to Ireland to provide clear and effective cost information to patients. It does however remain unclear how best to communicate such information to patients who wish to be informed. Furthermore, exploring the potential means by which such information could be communicated was not an aim of our study. Further cross-sectional research is needed to explore patient perspectives on how best to supply patients with this information. In addition, male patients with breast cancer did not participate in our study. Furthermore, we achieved relatively low levels of participation from patients with metastatic disease as compared to other research in this area [20]. Future research should aim to involve these patient groups. This future research may benefit from a qualitative approach due to sensitivity of such discussions and the low numbers of male patients with breast cancer.

Additionally, majority of patients found several cost-reduction methods to be acceptable. These cost-reduction methods included general principles and strategies towards healthcare policy which could be applied in a number of healthcare systems worldwide, e.g., increased drug cost transparency or increased governmental control of drug costs. Transparency in pricing has frequently been highlighted as an integral part of efforts to lower drug costs [28]. In our study, increased drug cost transparency was the most popular cost-reduction method, which highlights the acceptability of this cost-reduction method to patients.

Finally, a significant limitation of this study is the absence of multivariable-adjusted models to control for confounding. The aims of this study are observational and hypothesis-generating. Future research should aim to employ such statistical techniques to more closely explore factors associated with cost acceptability and patient desires to be better informed of cancer medication costs.

The issue of increasing costs of innovative anticancer medications is a growing one that affects low-, middle- and high-income countries around the world to varying extents and can limit access to new and effective treatments. For example, in Morrocco, 22 out of 39 innovative anticancer drugs that received market authorization were not reimbursed [29]. The United States has many of the highest absolute costs of anticancer medications when compared to other countries. However, when accounting for per capitata spending power, anticancer drugs are least affordable in poorer countries, for example India and China [30]. The limited availability and affordability of anticancer drugs give rise to substantial differences in cancer survival between high-income and low- and middle-income countries [22]. As such, it is necessary for future research to evaluate cost-reduction methods such as those described in this paper and to identify novel techniques to improve patient access to innovative medications.

## 5. Conclusions

The rapidly growing costs of cancer care present a significant threat to the sustainability of healthcare systems. Our findings highlight that patients find many societal oncology medication costs to be unacceptable. Our results also indicate that reducing the societal costs of cancer treatment is important to many patients. Furthermore, we identify several cost-reduction methods which are acceptable to a majority of patients.

## Figures and Tables

**Table 1:** Demographics of participants.

Participant Demographics		(Total Population N = 180)
**Mean Age in Years (Range)**		55.2 (24–83)
**Education Level N (%)**	Any Primary	6 (3.3%)
	Any Secondary	55 (30.6%)
	Diploma/Certificate	52 (28.9%)
	Primary University Degree	30 (16.7%)
	Postgraduate Degree	37 (20.6%)
**Yearly Income N (%)**	<€50,000	104 (57.8%)
	≥€50,000	76 (42.2%)
**Disease Stage N (%)**	Early stage (breast +/− lymph nodes)	161 (89.4%)
	Distant Metastases	19 (10.6%)
**Time From Diagnosis N (%)**	<1 year	33 (18.3%)
	1–3 years	67 (37.2%)
	4–5 years	26 (14.4%)
	>5 years	54 (30%)
**Treatment Received N (%)**	Medication	164 (91.1%)
	Surgery	160 (88.9%)
	Radiation Therapy	146 (81.1%)

**Table 2:** Chi-square and Mann–Whitney U tests for cost acceptability.

	Acceptable N (%)	Unacceptable N (%)	Test Statistic	Degrees of Freedom	*p*-Value
**Age**					
Median (IQR)	58 (52–68)	54 (49–61)	1861 ‡	-	**0.014**
**Education**					
No degree	12 (34.3%)	49 (33.8%)	0.003 †	1	0.956
Degree/diploma/certificate	23 (65.7%)	96 (66.2%)			
**Annual Household Income**					
<€50,000	21 (60.0%)	83 (57.2%)	0.088 †	1	0.767
≥€50,000	14 (40.0%)	62 (42.8%)			
**Time from Diagnosis**					
<1 year	2 (5.7%)	31 (21.4%)	5.280 †	2	0.071
1–5 years	19 (54.3%)	74 (51.0%)			
>5 years	14 (40.0%)	40 (27.6%)			
**Stage**					
Early stage	27 (77.1%)	134 (92.4%)		1	**0.014**
Metastatic	8 (22.9%)	11 (7.6%)			
**Use of Expensive Medications**					
No	29 (82.9%)	121 (83.4%)	0.007 †	1	0.933
Yes	6 (17.1%)	24 (16.6%)			

“†” indicates a Chi-square test statistic and “‡” indicates a Mann–Whitney U test statistic. Where there is no symbol present, this indicates a Fischer’s exact test was used. Statistically significant associations have been highlighted in bold. Median and IQR were used for the Mann–Whitney U test, all other descriptive statistics are presented as frequencies and proportions. “Any primary” or “Any Secondary” were coded into the “No degree” category. “Diploma/certificate”, “Primary University Degree” or “Postgraduate Degree” were coded into the “Degree/diploma/certificate” category.

**Table 3:** Chi-square and Mann–Whitney U tests for desire to be better informed of societal costs.

	Wish to Be Informed of Costs N (%)	Do Not Wish to Be Informed of Costs N (%)	Test Statistic	Degrees of Freedom	*p*-Value
**Age**					
Median (IQR)	55 (49–63)	54 (47.5–58.5)	2938 ‡	-	0.383
**Education**					
No degree	52 (39.7)	9 (18.4)	7.240 †	1	**0.007**
Degree/diploma/certificate	79 (60.3)	40 (81.6)			
**Annual Household Income**					
<€50,000	80 (61.1)	24 (49.0)	2.136 †	1	0.144
≥€50,000	51 (38.9)	25 (51.0)			
**Time from Diagnosis**					
<1 year	23 (17.6)	10 (20.4)	0.607 †	2	0.738
1–5 years	70 (53.4)	23 (46.9)			
>5 years	38 (29.0)	16 (32.7)			
**Stage**					
Early stage	122 (93.1)	39 (79.6)	6.923 †	1	**0.009**
Metastatic	9 (6.9)	10 (20.4)			
**Use of Expensive Medications**					
No	109 (83.2)	41 (83.7)	0.006 †	1	0.940
Yes	22 (16.8)	8 (16.3)			

“†” indicates a Chi-square test statistic and “‡” indicates a Mann–Whitney U test statistic. Where there is no symbol present, this indicates a Fischer’s exact test was used. Statistically significant associations have been highlighted in bold. Median and IQR were used for the Mann–Whitney U test, all other descriptive statistics are presented as frequencies and proportions. “Any primary” or “Any Secondary” were coded into the “No degree” category. “Diploma/certificate”, “Primary University Degree” or “Postgraduate Degree” were coded into the “Degree/diploma/certificate” category.

## Data Availability

The datasets presented in this article are not readily available because of commitments made in the original ethical approval request which prohibits sharing of the data for use by those outside of the study authors. Requests to access the datasets should be directed to Matthew Cronin.

## Supplementary Materials

The following supporting information can be downloaded at: https://www.mdpi.com/article/10.3390/curroncol33030161/s1, Table S1: Drug cost calculations—intravenous-targeted and immunotherapy; Table S2: Drug cost calculations—oral therapy; Table S3: Drug cost calculations—chemotherapy; Table S4: Drug cost calculation assumptions; Text S1: Participant questionnaire.

## References

[1] Li M., Ka D., Chen Q. (2024). Disparities in availability of new cancer drugs worldwide: 1990–2022. BMJ Glob. Health.

[2] Caswell-Jin J.L., Sun L.P., Munoz D., Lu Y., Li Y., Huang H., Hampton J.M., Song J., Jayasekera J., Schechter C. (2024). Analysis of Breast Cancer Mortality in the US—1975 to 2019. JAMA.

[3] O’Reilly S., Luis I.V., Adam V., Razis E.D., Urruticoechea A., Arahmani A., Carrasco E., Chua B.H., Bliss J., Straehle C. (2025). Advancing equitable access to innovation in breast cancer. npj Breast Cancer.

[4] The IQVIA Institute (2024). The Global Use of Medicines 2024: Outlook to 2028.

[5] The IQVIA Institute (2024). Global Oncology Trends 2024: Outlook to 2028.

[6] Prior S., Scott R., Hennessy, Walker E. (2021). Review of High-Tech Drug Expenditure. Irish Government Economic and Evaluation Service (IGEES), Department of Public Expenditre, Infrastructure, Public Service Reform and Digitalisation. https://www.gov.ie/en/irish-government-economic-and-evaluation-service-igees/igees-publication/review-of-high-tech-drug-expenditure-2021/.

[7] Connors J. (2018). Health Budget Oversight and Management: Alignment of Health Budget and National Service Plan. Department of Public Expenditure and Reform. http://www.budget.gov.ie/.

[8] Agarwal A., Livingstone A., Karikios D.J., Stockler M.R., Beale P.J., Morton R.L. (2021). Physician-patient communication of costs and financial burden of cancer and its treatment: A systematic review of clinical guidelines. BMC Cancer.

[9] Panteli D., Arickx F., Cleemput I., Dedet G., Eckhardt H., Fogarty E., Gerkens S., Henschke C., Hislop J., Jommi C. (2016). Pharmaceutical regulation in 15 European countries review. Health Syst. Transit..

[10] Health Service Executive (2022). Drugs Payment Scheme. https://www2.hse.ie/services/schemes-allowances/drugs-payment-scheme/card/.

[11] van Dijk E.F., Coşkuntürk M., Zuur A.T., van der Palen J., van der Graaf W.T., Timmer-Bonte J.N., Wymenga A.N. (2016). Willingness to accept chemotherapy and attitudes towards costs of cancer treatment; A multisite survey study in the Netherlands. Neth. J. Med..

[12] Ramers-Verhoeven C.W., Geipel G.L., Howie M. (2013). New insights into public perceptions of cancer. Ecancermedicalscience.

[13] Lakdawalla D.N., Romley J.A., Sanchez Y., Maclean J.R., Penrod J.R., Philipson T. (2012). How cancer patients value hope and the implications for cost-effectiveness assessments of high-cost cancer therapies. Health Aff..

[14] Krammer R., Heinzerling L. (2014). Therapy preferences in melanoma treatment—Willingness to pay and preference of quality versus length of life of patients, physicians and healthy controls. PLoS ONE.

[15] Weiss J., Kirchberger M.C., Heinzerling L. (2020). Therapy preferences in melanoma treatment-Willingness to pay and preference of quality versus length of life of patients, physicians, healthy individuals and physicians with oncological disease. Cancer Med..

[16] Meropol N.J., Schrag D., Smith T.J., Mulvey T.M., Langdon R.M., Blum D., Ubel P.A., Schnipper L.E., American Society of Clinical Oncology (2009). American Society of Clinical Oncology guidance statement: The cost of cancer care. J. Clin. Oncol..

[17] Zafar S.Y., Chino F., Ubel P.A., Rushing C., Samsa G., Altomare I., Nicolla J., Schrag D., Tulsky J.A., Abernethy A.P. (2015). The utility of cost discussions between patients with cancer and oncologists. Am. J. Manag. Care.

[18] Rai A., Zheng Z., Zhao J., de Moor J.S., Ekwueme D.U., Yabroff K.R. (2020). Patient-Provider Discussions About Out-of-Pocket Costs of Cancer Care in the U.S. Am. J. Prev. Med..

[19] Lam A.B., Nipp R.D., Hasler J.S., Hu B.Y., Zahner G.J., Robbins S., Wheeler S.B., Tagai E.K., Miller S.M., Peppercorn J.M. (2024). National survey of patient perspectives on cost discussions among recipients of copay assistance. Oncologist.

[20] Irwin B., Kimmick G., Altomare I., Marcom P.K., Houck K., Zafar S.Y., Peppercorn J. (2014). Patient experience and attitudes toward addressing the cost of breast cancer care. Oncologist.

[21] Bullock A.J., Hofstatter E.W., Yushak M.L., Buss M.K. (2012). Understanding patients’ attitudes toward communication about the cost of cancer care. J. Oncol. Pract..

[22] Erfani P., Okediji R.L., Mulema V., Cliff E.R.S., Asante-Shongwe K., Bychkovsky B.L., Fadelu T. (2025). Advancing Global Pharmacoequity in Oncology. JAMA Oncol..

[23] World Health Organization (2018). Pricing of Cancer Medicines and Its Impacts.

[24] Steele C., Harrington J.M., O’Reilly S. (2025). Awareness of modifiable lifestyle risk factors and acceptability of secondary risk reduction services amongst Irish breast cancer survivors and oncology healthcare professionals. Breast.

[25] Kieran R., Coakley K., Macanovic B., Weadick C., Keogh R., Cooke K., Allen M., Higgins M., O’Reilly S. (2025). Cutting waste in cancer care: Acceptability of novel waste-reduction strategies in oral therapies amongst Irish oncology stakeholders. Ir. J. Med. Sci..

[26] Meisenberg B.R., Varner A., Ellis E., Ebner S., Moxley J., Siegrist E., Weng D. (2015). Patient Attitudes Regarding the Cost of Illness in Cancer Care. Oncologist.

[27] Haberecht K., Schnuerer I., Gaertner B., John U., Freyer-Adam J. (2015). The Stability of Social Desirability: A Latent Change Analysis. J. Pers..

[28] Anderer S. (2026). Health Care Plan Aims to Lower US Drug Costs and Increase Transparency. JAMA.

[29] Benhima N., Afani L., Fadli M.E., Essâdi I., Belbaraka R. (2024). Investigating the availability, affordability, and market dynamics of innovative oncology drugs in Morocco: An original report. Int. J. Equity Health.

[30] Prasad V., De Jesús K., Mailankody S. (2017). The high price of anticancer drugs: Origins, implications, barriers, solutions. Nat. Rev. Clin. Oncol..

