# Supplementary material for: Breast Cancer Patient Attitudes Towards Oncology Drug Costs in Ireland
Source: Curr Oncol. 2026 Mar 12;33(3):161. doi: 10.3390/curroncol33030161 (PMC13025230; doi:10.3390/curroncol33030161)
Supplement: Supplementary file 1 [file curroncol-33-00161-s001.zip › curroncol-4109309-supplementary.pdf]

Supplemental Documentation

Drug Cost Calculations

The drugs were selected as they satisfy at least one of the following criteria: they are first in class for the treatment of breast cancer as outlined by the British National Formulary (BNF) [31], they are licensed by the BNF for the treatment of breast cancer [32-34], they are listed under the ODMS/PCRS [35], or they are recommended by HSE/National Cancer Control Programme guidelines [36]. It is important to note that drug costs vary considerably by country and depend upon a variety of factors including anthropometric measurements and drug procurement costs [37]. A specialist oncology pharmacist and consultant medical oncologist reviewed and verified all drug cost calculations and dosing to ensure that prices were reflective of international trends.

Cost calculations were based on several key assumptions. Firstly, medication vial sharing was not assumed where possible. This simplifies drug cost calculation and is in keeping with previous analyses performed by the National Centre for Pharmacoeconomics [38]. Secondly, calculations requiring accurate body weight or body surface area (BSA) dosing were performed assuming an average body weight of 70 kg, height of 165 cm and BSA of 1.77m^2.

Table S1. Intravenous Targeted and Immunotherapy Cost Calculations.

Dosing Source	Cost Source	Drug Name	Dosing	Quantity per Vial (mg)	Vials Needed	Manufacturer	Cost Per Vial (€)	Cost Calculation Explanation for 1 Year of Treatment	Cost per Year (€)
From NCCP 204 [47]: Pertuzumab + Trastuzumab + Docetaxel	IPHA [39]	Pertuzumab	840mg for 1st dose.<br>420mg then every 3 weeks.	420	2 for first dose and 1 thereafter	Roche Products Ireland Ltd	2505.71	1x(Initial dose: 840mg (2 vials): 2 x €2505.71)<br>+<br>16x(Subsequent Dose: 420mg (1 vial):<br>1 x €2505.71)<br>+<br>= €45102.78	45102.78
NA	D'Arpino Paper [45]	Trastuzumab (Kanjinti)	NA<br><br>Paper did not explain exact dosing, just cost for 17 treatment cycles using 420 mg vials.	420	NA	Amgen Ltd	NA	17 Doses	8499
From NCCP 688 [48]: Atezolizumab + Nab-paclitaxel	IPHA [39]	Atezolizumab	840mg on days 1 and 15 of 28 day cycle.<br>Until disease progression or toxicity	840	1	Roche Products Ireland Ltd	2980.35	2 doses every 28 days = 26 per year<br>26 x €2980.35 = €77489.1	77489.1
Martindale [49]	IPHA [39]	Pembrolizumab	200mg once every 3 weeks	100	2	Merck Sharp & Dohme Irl (Human Health) Ltd	3153.86	200 mg once every 3 weeks = 17 doses per year =<br>17 x 2(100mg vials) x €3153.86 = €107231.24	107231.24

Table S2. Oral Therapy Cost Calculations.

Dosing Source	Cost Source	Drug Name	Manufacturer	Cost per Packet (€)	Number of Tablets per Pack	Dosing per Tablet	Approximate Packs Required for 1 Year	Cost per Year
MIMS [40]	MIMS [40]	Tamoxifen	Rowex Ltd.	4.24	30	20 mg once daily	12	50.88
Martindale [50]	MIMS [41]	Anastrozole	Clonmel Healthcare Ltd.	13.44	28	1 mg once daily	13	174.72
Martindale [51]	PCRS [46]	Palbociclib	Pfizer	2365.2	21	125 mg once daily for 21 days with 7 day rest	13	30747.6

Table S3. Chemotherapy Cost Calculations.

Dosing Source	Cost Source	Drug Name	Dosing Formula (mg/m2)	Dose for given BSA (mg)	Dose Banding (mg)	Vials Needed	Manufacturer	Cost Calculation Explanation	Gross Dose Cost (£)	Gross Dose Cost (€)	Dosing Schedule	Cost for Treatment Regimen (€)
From NCCP 316 [36]: AC Protocol	BNF [44]	Paclitaxel	175	309.75	318	1 x (300mg) and 1 x (30mg)	Seacross Pharmaceuticals	£192.5 (300mg vial) + £35 (30mg vial)	227.5	266.175	Every 2 weeks for 4 cycles = 4 x €266.175 = €1064.70	1064.7
From NCCP 316 [36]: AC Protocol	MIMS [42]	Doxorubicin	60	106.2	110	2 x (50mg) + 1 x (10mg)	Hospira Ireland Sales Ltd	2 x €94.71 (50mg vial) + €19.20 (10mg vial)		208.62	Every 2 weeks for 4 cycles = 4 x €208.62 = €834.48	834.48
From NCCP 316 [36]: AC Protocol	MIMS [43]	Cyclophosphamide	600	1062	1100	1 x (1g) and 1 x (500mg)	Baxter Healthcare Ltd	€28 (1g vial) + €14 (500mg vial)		42	Every 2 weeks for 4 cycles = 4 x €42 = €168	168
From NCCP 250 [52]: Docetaxel + Cyclophosphamide	BNF [34]	Docetaxel	75	132.75	132	1 x (160mg)	Seacross Pharmaceuticals	£101.25 (160 mg vial)	101.25	118.4625	Every 3 weeks for 4 cycles 4 x €118.46 = €473.85	473.85

Table S4. Assumptions of Calculations Summarised.

Height (cm)	165
Body Weight (kg)	70
Body Surface Area (Du Bois Formula)	1.77
GBP EUR Exchange Rate	1.17
Assume no vial sharing	

Participant Invitation Letter

Text S1. Participant Questionnaire – Both versions of the Participant Invitation Letters with associated Participant Information Leaflets distributed at the clinical sites are also included.

My name is Matthew Cronin, I am a Medical Student at University College Cork.

I wish to carry out a project to assess **patient attitudes and awareness of the costs of oncology medications to society**.

The registered title of my project is: **"How much does your medication cost?": Assessing patient attitudes and awareness of the costs of oncology medication to society.**

My project supervisor is Professor Seamus O'Reilly, Consultant Medical Oncologist, Cork University Hospital.

I would like your help as we hope this project would guide further research to increase patient involvement in medication cost discussions.

Should you wish to take part you will be asked to fill out a questionnaire which should take approximately 10 minutes to complete.

Your participation is voluntary, and you are free to withdraw at any time when completing the questionnaire.

Should you decide to participate, the information gathered as part of this study will be used only in this study. No identifiable information about you will be used in the analysis. Your consent will be implied by completion and return of the questionnaire.

Thank you for your help in this matter.

If you are still interested in taking part, please read the participant information leaflet.

UCC
Coláiste na hOllscoile Corcaigh
University College Cork, Ireland

Participant Information Leaflet

Project Title: "How much does your medication cost?": Assessing patient attitudes and awareness of the costs of oncology medication to society.

Chief Investigator: Professor Seamus O'Reilly, Consultant Medical Oncologist, Cork University Hospital.
Contact Number for Chief Investigator: 021 4546400.

Co-investigator: Dr Ruth Kieran, Specialist Registrar in Medical Oncology, Cork University Hospital.

Co-investigator: Ms Katie Cooke, Specialist Oncology Pharmacist, Cork University Hospital.

Medical Student: Matthew Cronin, 4th Year Direct Entry Medical Student, University College Cork.
Email Address: 118475496@umail.ucc.ie.

1. Project Description

- The Irish healthcare system, unlike many others around the world has several schemes in place to **subsidise medication costs for patients**.
- For many of these schemes, the maximum out-of-pocket cost for patients is **capped at a fixed amount.** This means that **any additional costs over the price cap is paid by the healthcare system**.
- This has greatly **increased patient access** to newer and more effective treatments and has shifted the cost from the patient to the healthcare system.
- As a result, it has **decreased patient exposure to many of the high costs of oncology medications**.
- By conducting this study, we hope to better understand **patient attitudes and awareness of the true costs** of oncology medications to **<u>society/the healthcare system</u>**.

2. Why have I been chosen?

- You have been asked to participate as you are attending the breast cancer care clinic at Cork University Hospital.
- The outcomes of this study have the potential to affect medication cost discussions at a national level and could increase patient involvement in the discussions surrounding medication costs.

3. What will it involve?

- You will be asked to complete a questionnaire. It should take approximately **10 minutes** to complete.
- The questionnaire has **3 sections**. The first section will ask you **basic non-identifiable demographic questions like your age and diagnosis**.
- The second section will ask you about your **personal attitudes towards medication cost management**.
- The final section of the questionnaire will **provide you with specific costs for several oncology medications** used in the treatment of breast cancer. **You will be asked to indicate your attitudes towards these costs.**
- Once you have completed the questionnaire, please return it to the relevant medical professional from whom you received it.
- **Do not provide any personal identifying information** other than what is specifically requested in the questionnaire, i.e. **<u>do not include your name or medical record number etc</u>**.

UCC
Coláiste na hOllscoile Corcaigh
University College Cork, Ireland

4. What will happen to the information collected?

- Physical copies of the questionnaire response will be stored securely in the Department of Medical Oncology at Cork University Hospital for a short period and will be disposed of once transferred onto a secure electronic database.
- Your data will be stored on a password protected computer accessible only to the research team. The data will be retained for a maximum period of 10 years and then destroyed. Your information will be used in this study only.

5. Will the information I give be confidential?

- The data from this project will be analysed and presented in the medical student's final year research project report.
- No personally identifiable information to you, will be analysed or included in the findings.
- If an opportunity arose to publish this data in a research journal, again all personal details would be kept anonymous.
- Hard copies of questionnaires will be stored securely in the Chief Investigator's office in the Department of Medical Oncology & destroyed once uploaded into electronic format.

6. Support Services

- If you are affected by any of the questions or topics asked in this questionnaire, the following support services are available: The Irish Cancer Society, The Marie Keating Foundation, Europa Donna Ireland, The Irish Cancer Prevention Network and Cork Arc Cancer Support.

This study has been approved by the Clinical Research Ethics Committee of the Cork Teaching Hospitals, Lancaster Hall, 6 Little Hanover Street, Cork, 021 4901901.

Participant Invitation Letter

My name is Matthew Cronin, I am a Medical Student at University College Cork.

I wish to carry out a project to assess **patient attitudes and awareness of the costs of oncology medications to society**.

The registered title of my project is: **"How much does your medication cost?": Assessing patient attitudes and awareness of the costs of oncology medication to society.**

My project supervisor is Professor Seamus O'Reilly, Consultant Medical Oncologist, Cork University Hospital.

I would like your help as we hope this project would guide further research to increase patient involvement in medication cost discussions.

Should you wish to take part you will be asked to fill out a questionnaire which should take approximately 10 minutes to complete.

Your participation is voluntary, and you are free to withdraw at any time when completing the questionnaire.

Should you decide to participate, the information gathered as part of this study will be used only in this study. No identifiable information about you will be used in the analysis. Your consent will be implied by completion and return of the questionnaire.

Thank you for your help in this matter.

If you are still interested in taking part, please read the participant information leaflet.

Participant Information Leaflet

Project Title: "How much does your medication cost?": Assessing patient attitudes and awareness of the costs of oncology medication to society.

Chief Investigator: Professor Seamus O'Reilly, Consultant Medical Oncologist, Cork University Hospital.
Contact Number for Chief Investigator: 021 4546400.

Co-investigator: Dr Ruth Kieran, Specialist Registrar in Medical Oncology, Cork University Hospital.

Co-investigator: Ms Katie Cooke, Specialist Oncology Pharmacist, Cork University Hospital.

Medical Student: Matthew Cronin, 4th Year Direct Entry Medical Student, University College Cork.
Email Address: 118475496@umail.ucc.ie.

1. Project Description

- The Irish healthcare system, unlike many others around the world has several schemes in place to **subsidise medication costs for patients**.
- For many of these schemes, the maximum out-of-pocket cost for patients is **capped at a fixed amount.** This means that **any additional costs over the price cap is paid by the healthcare system**.
- This has greatly **increased patient access** to newer and more effective treatments and has shifted the cost from the patient to the healthcare system.
- As a result, it has **decreased patient exposure to many of the high costs of oncology medications**.
- By conducting this study, we hope to better understand **patient attitudes and awareness of the true costs** of oncology medications to **society/the healthcare system**.

2. Why have I been chosen?

- You have been asked to participate as you are attending the breast cancer care clinic at either South Infirmary Victoria University Hospital or Consultants Private Clinic.
- The outcomes of this study have the potential to affect medication cost discussions at a national level and could increase patient involvement in the discussions surrounding medication costs.

3. What will it involve?

- You will be asked to complete a questionnaire. It should take approximately **10 minutes** to complete.
- The questionnaire has **3 sections**. The first section will ask you **basic non-identifiable demographic questions like your age and diagnosis**.
- The second section will ask you about your **personal attitudes towards medication cost management**.
- The final section of the questionnaire will **provide you with specific costs for several oncology medications** used in the treatment of breast cancer. **You will be asked to indicate your attitudes towards these costs.**
- Once you have completed the questionnaire, please return it to the relevant medical professional from whom you received it.
- **Do not provide any personal identifying information** other than what is specifically requested in the questionnaire, i.e. **do not include your name or medical record number etc**.

4. What will happen to the information collected?

- Physical copies of the questionnaire response will be stored securely in the South Infirmary Victoria University Hospital/Consultants Private Clinic outpatient department for a short period and will be disposed of once transferred onto a secure electronic database.
- Your data will be stored on a password protected computer accessible only to the research team. The data will be retained for a maximum period of 10 years and then destroyed. Your information will be used in this study only.

5. Will the information I give be confidential?

- The data from this project will be analysed and presented in the medical student's final year research project report.
- No personally identifiable information to you, will be analysed or included in the findings.
- If an opportunity arose to publish this data in a research journal, again all personal details would be kept anonymous.
- Hard copies of questionnaires will be stored securely in the Chief Investigator's office in the Department of Medical Oncology & destroyed once uploaded into electronic format.

6. Support Services

- If you are affected by any of the questions or topics asked in this questionnaire, the following support services are available: The Irish Cancer Society, The Marie Keating Foundation, Europa Donna Ireland, The Irish Cancer Prevention Network and Cork Arc Cancer Support.

This study has been approved by the Clinical Research Ethics Committee of the Cork Teaching Hospitals, Lancaster Hall, 6 Little Hanover Street, Cork, 021 4901901.

Participant Questionnaire

Study Title: "How much does your medication cost?": Assessing patient attitudes and awareness of the costs of oncology medication to society.

Chief Investigator: Professor Seamus O'Reilly, Consultant Medical Oncologist, Department of Medical Oncology, Cork University Hospital, Wilton, Cork.

Medical Student: Matthew Cronin, Direct Entry Medicine, Year 4.

Overview

Thank you for taking the time to complete this questionnaire. It should take approximately 10 minutes to complete. If you have any questions about the content of this questionnaire, please feel free to contact one of the researchers using the details provided in the information leaflet.

<u>**Please answer all questions, even if you have not been treated with any of the medications listed**</u>. Please <u>**carefully read the overview**</u> of the three sections of the questionnaire outlined below:

Section 1 – Demographics

- This section will ask **general non-identifiable demographic questions** about yourself such as your **age and cancer diagnosis**.

Section 2 – Attitudes to Medication Cost Management

- This section will ask about **your attitudes towards oncology medication cost management**.

Section 3 – Attitudes and Awareness of Oncology Medication Costs

- <u>**Please answer all questions in this section, even if you have not been treated with any of the medications listed**</u>.
- The final section of this questionnaire will provide a **list of commonly prescribed medications** used in the treatment of breast cancer.
- Information will be provided regarding how the medication is administered.
- Then the **average cost to <u>the healthcare system/society</u>** required to supply this medication for a given duration is shown.
- You will then be asked to **indicate your attitudes towards these costs**.

Section 1: Demographics

1. What age are you? (Years)

____

2. What is your gender?

☐ Male.

☐ Female.

☐ Prefer not to say.

3. What is the highest level of education you have completed to date?

☐ Some primary (not complete).

☐ Primary or equivalent.

☐ Inter/Junior Certificate or equivalent.

☐ Leaving Certificate or equivalent

☐ Diploma/cert.

☐ Primary University Degree.

☐ Postgraduate/higher Degree

4. Please indicate your current level of employment.

☐ Full-time employment.

☐ Part-time employment.

☐ Retired.

☐ Homemaker.

☐ Disabled from cancer.

☐ Disabled from another cause.

☐ Student.

☐ Unemployed.

5. What is your average yearly household income?

☐ Less than €15,000.

☐ Between €15,000 and €29,999.

☐ Between €30,000 and €49,999.

☐ Between €50,000 and €99,999.

☐ More than €100,000.

6. Which of the following best describes your cancer stage?

☐ Breast Cancer, limited to the breast and lymph nodes on the same side.

☐ Breast Cancer, spread to other parts of your body (i.e. lung, liver or bone etc).

7. What year were you first diagnosed with breast cancer?

____

8. Have you been diagnosed with more than one cancer?

☐ If yes, please specify the other diagnosis.

____

☐ No.

9. Have you had a friend or family member who has suffered from cancer in the past?

☐ Yes.

☐ No.

10. Treatment received ***(please tick all that are applicable)***

☐ I have been prescribed **medication** to treat my cancer (i.e. tablets, injections, infusions, etc).

☐ I have had **surgery** to treat my breast cancer.

☐ I have received **radiation therapy** to treat my breast cancer.

☐ I have not received any treatment for my breast cancer yet.

☐ Not sure.

Section 2: Attitudes to Medication Cost Management

11. Please indicate to what extent you agree or disagree with the following statements.
(Please tick one box for each statement).

	Strongly Agree	Agree	Neither Agree nor Disagree	Disagree	Strongly Disagree
I understand the costs that I, personally, will pay for my cancer treatment.	☐	☐	☐	☐	☐
I have been surprised by the costs to me personally of my cancer treatment.	☐	☐	☐	☐	☐
I understand the costs that society will pay for my cancer treatment.	☐	☐	☐	☐	☐
I am aware that the costs I personally pay for my cancer care are different to the costs paid by society.	☐	☐	☐	☐	☐
I believe the cost to society of cancer care is high.	☐	☐	☐	☐	☐
I would like to be better informed of the costs to society of my cancer treatment.	☐	☐	☐	☐	☐
I think reducing the costs of cancer care to society is important.	☐	☐	☐	☐	☐

12. How do you think we could reduce the costs of cancer care to society?
(Please tick all boxes that you agree with).

☐ Increase government control of the costs of cancer medications.
☐ Use the least expensive treatment when there are two good options available.
☐ Improve awareness of medication costs among doctors.
☐ Improve awareness of medication costs among patients.
☐ Improve awareness of medication costs among the public.
☐ Require medication companies to be more transparent about medication costs.
☐ Involve patients in discussions of medication costs at national and international levels.
☐ Increase competition between medication companies.
☐ Manufacture more cancer medications in Ireland.
☐ Pay doctors less.
☐ Use more nurses instead of doctors.
☐ I don’t think we should care about lowering the cost to society of cancer care.
☐ Other (Please specify):

____

Section 3: Attitudes and Awareness of Medication Costs

In this final section we have provided **3 lists of medications** commonly prescribed in the care of patients diagnosed with breast cancer. There are **11 medications in total**.

<u>Please answer all questions even if you have not received treatment with all the medications described.</u>

Below we give a brief explanation of **how each medication is administered** and the **approximate cost to society/the healthcare system** to provide the medication for a given duration. We ask that you read each question carefully and **indicate your attitudes towards the costs by ticking the appropriate boxes**.

Where possible the **brand name has been indicated in brackets**.

Where accurate bodyweight or body surface area dosing is required, an average bodyweight of 70kg and height of 165cm is used. Costs shown are based solely on the individual cost of the medication and does not include additional costs such as labour or equipment.

List 1

Tamoxifen

This is a **tablet medication taken orally once daily**. It comes in packets of 30 tablets.

It costs the healthcare system **approximately €51** to provide this medication for **one patient per year**.

13. I have been treated with this medication before: Yes ☐ No ☐ Not sure ☐

14. Please indicate your attitude to this cost below. ***(Please tick just one box).***

☐ This cost is much less than I expected.
☐ This cost is less than I expected.
☐ This cost is as I expected.
☐ This cost is more than I expected.
☐ This cost is much more than I expected.

15. Do you believe this is an acceptable cost for this medication?

☐ Yes.
☐ No.

Anastrozole

This is a **tablet medication taken orally once daily**. It comes in packets of 28 tablets.

It costs the healthcare system **approximately €175** to provide this medication for **one patient per year**.

16. I have been treated with this medication before: Yes ☐ No ☐ Not sure ☐

17. Please indicate your attitude to this cost below. ***(Please tick just one box).***

☐ This cost is much less than I expected.
☐ This cost is less than I expected.
☐ This cost is as I expected.
☐ This cost is more than I expected.
☐ This cost is much more than I expected.

18. Do you believe this is an acceptable cost for this medication?

☐ Yes.
☐ No.

Palbociclib (Ibrance)

This is a **tablet medication taken orally once daily** with food for three weeks followed by a rest week where no medication is taken. It comes in packets of 21 tablets.

It costs the healthcare system **approximately €30,748** to provide this medication for **one patient per year**.

19. I have been treated with this medication before: Yes ☐ No ☐ Not sure ☐

20. Please indicate your attitude to this cost below. ***(Please tick just one box).***

☐ This cost is much less than I expected.
☐ This cost is less than I expected.
☐ This cost is as I expected.
☐ This cost is more than I expected.
☐ This cost is much more than I expected.

21. Do you believe this is an acceptable cost for this medication?

☐ Yes.
☐ No.

List 2

Trastuzumab (Kanjinti)

This medication comes in vials and is **injected slowly into a vein** over a 30-minute period. It is usually given **once every three weeks** for 17 cycles of treatment.

It costs the healthcare system **approximately €8,499** to provide this medication for **one patient per year**.

22. I have been treated with this medication before: Yes ☐ No ☐ Not sure ☐

23. Please indicate your attitude to this cost below. ***(Please tick just one box).***

☐ This cost is much less than I expected.
☐ This cost is less than I expected.
☐ This cost is as I expected.
☐ This cost is more than I expected.
☐ This cost is much more than I expected.

24. Do you believe this is an acceptable cost for this medication?

☐ Yes.
☐ No.

Pertuzumab (Perjeta)

This medication comes in vials and is **injected slowly into a vein** over a 30 to 60-minute period. It is usually **given once every three weeks** for 17 cycles of treatment.

It costs the healthcare system **approximately €45,103** to provide this medication for **one patient per year**.

25. I have been treated with this medication before: Yes ☐ No ☐ Not sure ☐

26. Please indicate your attitude to this cost below. ***(Please tick just one box).***

☐ This cost is much less than I expected.
☐ This cost is less than I expected.
☐ This cost is as I expected.
☐ This cost is more than I expected.
☐ This cost is much more than I expected.

27. Do you believe this is an acceptable cost for this medication?

☐ Yes.

☐ No.

Atezolizumab (Tecentriq)

This medication comes in vials and is **injected slowly into a vein** over a 60-minute period. It is usually given **once every two weeks**.

It costs the healthcare system **approximately €77,489** to provide this medication for **one patient per year**.

28. I have been treated with this medication before: Yes ☐ No ☐ Not sure ☐

29. Please indicate your attitude to this cost below. *(Please tick just one box).*

☐ This cost is much less than I expected.
☐ This cost is less than I expected.
☐ This cost is as I expected.
☐ This cost is more than I expected.
☐ This cost is much more than I expected.

30. Do you believe this is an acceptable cost for this medication?

☐ Yes.
☐ No.

Pembrolizumab (Keytruda)

This medication comes in vials and is **injected slowly into a vein** over a 30-minute period. It is usually given **once every three weeks** for 17 cycles of treatment.

It costs the healthcare system **approximately €107,231** to provide this medication for **one patient per year**.

31. I have been treated with this medication before: Yes ☐ No ☐ Not sure ☐

32. Please indicate your attitude to this cost below. *(Please tick just one box).*

☐ This cost is much less than I expected.
☐ This cost is less than I expected.
☐ This cost is as I expected.
☐ This cost is more than I expected.
☐ This cost is much more than I expected.

33. Do you believe this is an acceptable cost for this medication?

☐ Yes.
☐ No.

List 3

Cyclophosphamide

This medication comes in vials and is **injected slowly into a vein** over a 30-minute period. It is usually given **once every two weeks** for 4 cycles of treatment, lasting 8 weeks.

It costs the healthcare system **approximately €168** to provide this medication for **one patient for a full treatment course.**

34. I have been treated with this medication before: Yes ☐ No ☐ Not sure ☐

35. Please indicate your attitude to this cost below. ***(Please tick just one box).***

☐ This cost is much less than I expected.
☐ This cost is less than I expected.
☐ This cost is as I expected.
☐ This cost is more than I expected.
☐ This cost is much more than I expected.

36. Do you believe this is an acceptable cost for this medication?

☐ Yes.
☐ No.

Docetaxel

This medication comes in vials and is **injected slowly into a vein** over a 60-minute period. It is usually given **once every three weeks** for 4 cycles of treatment, lasting 12 weeks.

It costs the healthcare system **approximately €474** to provide this medication for **one patient for a full treatment course.**

37. I have been treated with this medication before: Yes ☐ No ☐ Not sure ☐

38. Please indicate your attitude to this cost below. ***(Please tick just one box).***

☐ This cost is much less than I expected.
☐ This cost is less than I expected.
☐ This cost is as I expected.
☐ This cost is more than I expected.
☐ This cost is much more than I expected.

39. Do you believe this is an acceptable cost for this medication?

☐ Yes.
☐ No.

Doxorubicin

This medication comes in vials and is **injected slowly into a vein** over a 15-minute period. It is usually given **once every two weeks** for 4 cycles of treatment, lasting 8 weeks.

It costs the healthcare system **approximately €834** to provide this medication for **one patient for a full treatment course.**

40. I have been treated with this medication before: Yes ☐ No ☐ Not sure ☐

41. Please indicate your attitude to this cost below. ***(Please tick just one box).***

- ☐ This cost is much less than I expected.
- ☐ This cost is less than I expected.
- ☐ This cost is as I expected.
- ☐ This cost is more than I expected.
- ☐ This cost is much more than I expected.

42. Do you believe this is an acceptable cost for this medication?

- ☐ Yes.
- ☐ No.

Paclitaxel

This medication comes in vials and is **injected slowly into a vein** over a three-hour period. It is usually given **once every two weeks** for 4 cycles of treatment, lasting 8 weeks.

It costs the healthcare system **approximately €1,065** to provide this medication for **one patient for a full treatment course**.

43. I have been treated with this medication before: Yes ☐ No ☐ Not sure ☐

44. Please indicate your attitude to this cost below. ***(Please tick just one box).***

- ☐ This cost is much less than I expected.
- ☐ This cost is less than I expected.
- ☐ This cost is as I expected.
- ☐ This cost is more than I expected.
- ☐ This cost is much more than I expected.

45. Do you believe this is an acceptable cost for this medication?

- ☐ Yes.
- ☐ No.

Final question on the next page →

46. Please tell us to what extent you agree or disagree with the following statements.
(Please tick one box for each statement).

	Strongly Agree	Agree	Neither Agree nor Disagree	Disagree	Strongly Disagree
Overall, I was surprised by the costs presented in this questionnaire.	☐	☐	☐	☐	☐
Overall, I felt the costs discussed in this questionnaire were higher than I expected.	☐	☐	☐	☐	☐
Overall, I felt the costs discussed in this questionnaire were acceptable.	☐	☐	☐	☐	☐
After completing this questionnaire, I think reducing the cost to society of cancer care is important.	☐	☐	☐	☐	☐
After completing this questionnaire, I would like to be better informed of the costs to society of my cancer treatment.	☐	☐	☐	☐	☐

Thank you for taking the time to complete this questionnaire.
Your response is greatly appreciated.
Please return the questionnaire to the healthcare professional from whom you received it.

References

31. Digital Medicines Information Suite. Medicines Complete. Available online: https://www.medicinescomplete.com/#/content/bnf/_159392432?hspl=breast&hspl=cancer (accessed on).
32. MedicinesComplete—Log in [Internet]. www.medicinescomplete.com. Available online: https://www.medicinescomplete.com/#/content/bnf/_988968089?hspl=pembrolizumab (accessed on).
33. MedicinesComplete—Log in [Internet]. www.medicinescomplete.com. Available online: https://www.medicinescomplete.com/#/content/bnf/_938546664?hspl=doxorubicin (accessed on).
34. MedicinesComplete—Log in [Internet]. www.medicinescomplete.com. Available online: https://www.medicinescomplete.com/#/content/bnf/_904039742?hspl=docetaxel (accessed on).
35. Health Service Executive. *Cancer Drugs Approved for Reimbursement*; Cancer Drugs Approved for Reimbursement—HSE.ie; Health Service Executive: National Cancer Control Program: Dublin, Ireland, 2023.
36. NCCP National SACT Regimen. Available online: https://www.hse.ie/eng/services/list/5/cancer/profinfo/chemoprotocols/breast/316-dose-dense-doxorubicin-cyclophosphamide-ac-60-600-14-day-followed-by-paclitaxel-175-14-day-and-trastuzumab-therapy-dd-ac-th-.pdf (accessed on).
37. Simoens, S.; Vulto, A.G.; Dylst, P. Simulating Costs of Intravenous Biosimilar Trastuzumab vs. Subcutaneous Reference Trastuzumab in Adjuvant HER2-Positive Breast Cancer: A Belgian Case Study. *Pharmaceuticals* **2021**, *14*, 450. https://doi.org/10.3390/ph14050450.
38. National Centre for Pharmacoeconomics. *Cost Effectiveness of Pertuzumab (Perjeta®) in Combination with Trastuzumab and Docetaxel in Adults with HER2-Positive Metastatic or Locally Recurrent Unresectable Breast Cancer Who Have Not Received Previous Anti-HER2 Therapy or Chemotherapy*; National Centre for Pharmacoeconomics: Dublin, Ireland, 2013.
39. Irish Pharmaceutical Healthcare Association. *Price Realignment File for Implementation on 1 March 2023*; Irish Pharmaceutical Healthcare Association: Dublin, Ireland, 2023.
40. TAMOX: [Internet]. MIMS Ireland. Available from: TAMOX: MIMS Ireland.
41. AMIDEX: [Internet]. MIMS Ireland. Available from: AMIDEX: MIMS Ireland.
42. DOXORUBICIN: [Internet]. MIMS Ireland. Available from: DOXORUBICIN: MIMS Ireland.
43. ENDOXANA: [Internet]. MIMS Ireland. Available from: ENDOXANA: MIMS Ireland.
44. Digital Medicines Information Suite. MedicinesComplete. Available online: https://www.medicinescomplete.com/#/content/bnf/_465902208?hspl=paclitaxel (accessed on).
45. D'Arpino, A.; Savoia, M.; Cirillo, L.; Despiégel, N.; Haffemayer, B.; Giannopoulou, A.; Gaikwad, I. Pcn72 comparative cost analysis of subcutaneous trastuzumab originator (herceptin®) vs. intravenous trastuzumab biosimilar (kanjinti) from a hospital perspective in Italy. *Value Health* **2019**, *22*, S449.
46. Health Service Executive. *Hse—Primary Care Reimbursement Service Update to List of Agreed Prescribable Medicinal Products to Be Dispensed Under the High Tech Scheme Effective*; Health Service Executive: Dublin, Ireland, 2022; p. 111.
47. Hennessy, B.; Keane, M. NCCP National SACT Regimen. 2015. Available online: https://www.hse.ie/eng/services/list/5/cancer/profinfo/chemoprotocols/breast/204-pertuzumab-and-trastuzumab-and-docetaxel-therapy-21-day-cycle.pdf (accessed on).
48. NCCP Chemotherapy Regimen NCCP Regimen: Atezolizumab and Nab-Paclitaxel Therapy. 2022. Available online: https://www.hse.ie/eng/services/list/5/cancer/profinfo/chemoprotocols/breast/688.pdf (accessed on).
49. MedicinesComplete—Log in [Internet]. www.medicinescomplete.com. Available online: https://www.medicinescomplete.com/#/content/martindale/29515-q?hspl=pembrolizumab (accessed on).
50. MedicinesComplete—Log in [Internet]. www.medicinescomplete.com. Available online: https://www.medicinescomplete.com/#/content/martindale/14549-q?hspl=Anastrozole (accessed on).
51. MedicinesComplete—Log in [Internet]. www.medicinescomplete.com. Available online: https://www.medicinescomplete.com/#/content/martindale/29584-x?hspl=palbociclib (accessed on).
52. NCCP Chemotherapy Regimen NCCP Regimen: DOCEtaxel and Cyclophosphamide Therapy-21 day. 2015. Available online: https://www.hse.ie/eng/services/list/5/cancer/profinfo/chemoprotocols/breast/250-docetaxel-cyclophosphamide-tc-therapy-21-day.pdf (accessed on).
